# Focal Segmental Glomerulosclerosis and Scheduled Pretransplant Plasmapheresis: A Timely Diagnosis of Nail-Patella Syndrome Avoided More Futile Immunosuppression

**DOI:** 10.1155/2020/8879555

**Published:** 2020-07-24

**Authors:** H. Trimarchi

**Affiliations:** Nephrology Service, Hospital Británico de Buenos Aires, Buenos Aires, Argentina

## Abstract

Focal and segmental glomerulosclerosis (FSGS) is a histopathological pattern of injury. As such, it encompasses a wide variety of dissimilar entities with different pathophysiologic mechanisms. Although ultrastructural morphological characteristics can specifically diagnose certain diseases and genetic mutations can also be unravelled, this ideal situation is generally not available worldwide. In this respect, when proteinuria with or without nephrotic syndrome is encountered and FSGS is the histological lesion, patients start to be prescribed different regimes of immunosuppression, which should only be indicated in cases of primary FSGS, a rare entity that is elusive to response and can hardly be precisely diagnosed. We present a 35-year-old female patient with a life-long diagnosis of FSGS and a heavy burden of immunosuppressants, which had been unable to manage the persistent proteinuria that eventually led to end-stage kidney disease. She was referred to us to organize the kidney transplant. Plasmapheresis had been previously suggested to her to prevent the relapse of primary FSGS. A genetic test disclosed that the patient was heterozygous for LMX1B, and the diagnosis of nail-patella syndrome was made. In this entity, immunosuppression is not indicated, and there is no recurrence of the disease in the transplanted allograft.

## 1. Introduction

Focal and segmental glomerulosclerosis (FSGS) is not a disease. It is a histologic pattern of injury with several morphological variants that reflects the repairing mechanisms employed in response to past and/or present insults received by the kidney. It has been classically classified as primary or secondary [1, 2]. The origin of these insults is quite diverse. In the case of primary FSGS, it includes circulating factors mainly secreted by neutrophils or mononuclear cells that affect the podocyte. In secondary FSGS, it encompasses mutations of genes that codify podocyte proteins, hyperfiltration settings such as obesity, reduced nephron mass, vesical-ureteral reflux, drepanocytosis, certain drugs as biphosphonates and cocaine, and HIV, among others [3]. The clinical picture is generally more aggressive in primary FSGS, where nephrotic syndrome is prevalent and a progressive decline in glomerular filtration rate is faster than in secondary FSGS, where proteinuria is generally lower than the nephrotic range. Nephroprotection is indicated in both primary and secondary FSGS. However, management of primary FSGS is mainly based on immunosuppression, with a discrete rate of successful response, while the secondary forms approach is mainly based on treating the primary cause [4]. Finally, approximately 30–55% of primary FSGS cases recur in the transplant with a high rate of graft loss, while most of secondary causes do not recur [5].

Genetic causes of FSGS due to mutations in podocyte proteins are frequently not diagnosed due to the lack of access to these genetic studies, and subdiagnosis exists. While some cases appear clinically soon after birth with severe nephrotic syndrome and renal failure, as it occurs with mutations in nephrin, other cases must be suspected early in childhood, as is the case of mutations in podocin, CD2-associated protein (CD2AP), and actinin-IV [6]. Finally, others manifest in later adolescence or early adulthood, as transient receptor potential cation channel subfamily C member 6 (TRPC6) and Wilms' tumor 1 (WT-1) [6]. The amount of proteinuria varies, generally increasing with age and reaching high levels, but nephrotic syndrome is not as frequent as in primary FSGS. In general, there are no pathognomonic features that determine the cause of FSGS in optic microscopy, while immunofluorescence is not contributory. Electron microscopy may be diagnostic in some cases and is not always available. Thus, the precise diagnosis of FSGS is mandatory, particularly to distinguish primary from genetic causes. In this work, we present a case that was assumed as primary FSGS for over 30 years of age, and the patient had been treated with different and aggressive immunosuppressive regimes with poor results. In fact, plasmapheresis was scheduled for the forthcoming peritransplant period. The patient was referred to our unit. A genetic study was asked, and the diagnosis of nail-patella syndrome was made due to a mutation in *LMX1B*. Immunosuppression has no place in the treatment of this cause of secondary FSGS.

## 2. Case Presentation

A 35-year-old female with a history of chronic proteinuria since early childhood was assessed due to end-stage kidney disease. She lacked a family history of chronic renal disease. At the age of 5 years, with a 2.9 g/day of proteinuria, she was empirically prescribed meprednisone 1 mg/kg/day for 12 weeks and, then, tapered down, without response. She was first biopsied at the age of 9 due to 3.9 g/day of proteinuria and normal kidney function without nephrotic syndrome. She was started on a low-protein and low sodium diet and enalapril at a dose of 5 mg twice daily. The kidney biopsy disclosed focal and segmental glomerulosclerosis and minimal interstitial fibrosis and tubular atrophy. Secondary causes were discarded, and since then, she had been prescribed different immunosuppression regimes that included steroids, cyclophosphamide, cyclosporine, and tacrolimus along her life. All the treatments failed to reduce proteinuria, which on the contrary, progressively increased until adulthood. Her glomerulosclerosis was filiated as primary. She was biopsied again at the age of 29 with a glomerular filtration rate of 55 mL/min and 6 g/day of proteinuria. The biopsy revealed 8 obliterated glomeruli plus focal and segmental glomerulosclerosis with segmental hyalinosis of the glomerular tuft in the remaining 9 glomeruli, plus a 40% of interstitial fibrosis. She was referred to our clinic at the age of 34 for a second opinion, as a kidney transplant had been counselled in another hospital, where plasmapheresis was to be performed as part of the induction therapy, with her father as the potential donor. We first indicated a genetic assessment of the glomerulosclerotic pattern, as the diagnosis of primary glomerulosclerosis appeared doubtful. The genetic study, performed with sequence analysis and copy number variation analysis of a panel of podocyte genes informed that the patient was heterozygous for *LMX1B* c.737G > A, p. (Arg246Gln), which was classified as pathogenic (Blueprint Genetics Oy, Keilaranta 16 A-B, 02150 Espoo, Finland). The molecular diagnosis of nail-patella syndrome was made. Knee and elbow X-Rays were normal, ophthalmologic evaluation discarded glaucoma, and nail features were unremarkable. The patient has recently been started on hemodialysis. She has recently received a kidney transplant from her father, in whom the genetic assessment had been previously made and the mutation was not encountered. The initial prescription of plasmapheresis as part of the induction approach to the transplant therapy was obviously discarded.

## 3. Discussion

In the present report, it is underscored that FSGS is a morphological pattern of injury that does not necessarily implie the necessity to employ immunosuppression despite elevated proteinuria, as commonly observed. The diagnosis of secondary causes of FSGS as genetic mutations is mandatory to undertake specific therapeutic indications and genetic counselling.

Focal and segmental glomerulosclerosis describes a distortion of the normal glomerular architecture characterized by a certain number of glomeruli with different degrees of sclerosis while others remain normal. The histological Columbia classification of FSGS identified five morphological variants of primary and secondary forms with dissimilar frequencies of diagnosis: (a) not otherwise specified form (NOS) or “classic FSGS” (68%of cases); (b) tip variant or tip lesion, with the sclerotic lesion next to the proximal tubule (10% of cases); (c) cellular variant with segmental endocapillary hypercellularity (3%); (d) perihilar variant, with perihilar hyalinosis and sclerosis in >50% of the glomeruli with segmental lesions (7%); and (e) collapsing variant, which requires at least a single glomerulus with a collapsing lesion, defined as segmental or global collapse of the tuft with overlying visceral epithelial cell hyperplasia (12%) [1, 2].

Segmental sclerosis lesions are consequently heterogeneous, and the different morphological variants may shed some light onto the many different pathogenetic pathways that share the unspecific morphology of FSGS. In this respect, the broad-based scars suggest previous healed necrotizing lesions; perihilar segmental sclerosis and hyalinosis are hallmarks of adaptive haemodynamic changes; visceral epithelial hyperplasia and hypertrophy and tip lesions are markers of podocyte injury, known as podocytopathies [2, 7].

Podocyte mutations can lead to FSGS and to alterations in the normal glomerular filtration barrier, clinically characterized by varying degrees of proteinuria and a progressive decrease in renal function [3, 6]. A rare cause of FSGS is the nail-patella syndrome.

The nail-patella syndrome or osteo-onychodysplasia that the reported patient presents is a rare autosomal dominant disorder with full gene penetrance but variability of expression within families, characterized by limb and pelvic skeletal abnormalities as hypoplastic or absent patella, dysplasia of elbows, iliac horns, nail and distal digital abnormalities, sensorineural hearing loss, ophthalmological abnormalities including glaucoma, gastrointestinal dysmotility, and kidney disease. The estimated incidence of nail-patella syndrome is estimated to be 1 per 50,000 [8–11].

Approximately 85 percent of families with nail-patella syndrome present with mutations of the *LMX1B* gene located at the distal end of the long arm of chromosome 9 [12]. LMX1B is a transcription factor of the LIM-homeodomain type that plays an important role for limb and renal development in vertebrates; however, it is expressed lifelong within the podocyte, and it is essential for the maintenance of the structured actin cytoskeleton in podocytes [13]. The identification of complete *LMX1B* deletions confirms that haploinsufficiency is the principal pathogenetic mechanism of nail-patella syndrome. Studies in homozygous knock-out mice and *in vitro* assays demonstrated that *LMX1B* protein expressed in glomerular podocytes helps control the transcription of multiple genes integral for proper glomerular basement membrane formation and/or glomerular podocyte differentiation and function during the early stages of renal development [12]. Putative target genes include *COL4A3* and *COLA4*, genes for alpha-4 chains of collagen type IV, and *NPHS2* and *CD2AP* genes, which encode podocyte proteins podocin and CD2-associated protein (CD2AP), respectively [14, 15].

In addition, there are case reports of *LMX1B* mutation (R246 or R249 missense mutations) associated with familial focal segmental glomerulosclerosis (FSGS), but without any other clinical features of nail-patella syndrome [16–18]. As in our patient, the kidney appears to be the only clinically affected organ, but her biological parents lacked the mutation, suggesting a spontaneous or *de novo* mutation. Renal biopsy is not necessary if the diagnosis is confirmed by genetic testing. However, if electron microscopy is performed, besides podocyte foot-process effacement, unique or pathognomonic changes in the kidney comprise focal or diffuse thickening of the glomerular basement membrane, with electron-lucent areas containing bundles of type III collagen alternating with areas of fibrillar inclusions in the glomerular basement membrane, giving a “moth-eaten appearance” [6]. In mice, the splitting of the glomerular basement membrane and the reduced number of endothelial fenestrations have been observed because podocytes synthesize proteins of the glomerular basement membrane and control differentiation of glomerular endothelial cells, as the vascular endothelial growth factor (VEGF) [6]. There is no clear relationship between the clinical course and renal histopathology [19], and findings on renal biopsy appear not to alter the clinical management [20, 21].

Renal involvement in nail-patella syndrome can be a significant threat to the quality of life in patients. Urinary abnormalities often manifest as asymptomatic proteinuria (20–30% of cases) or hematuria, but it can progress to end-stage renal disease, even during early childhood [10, 19]. Most affected individuals manifest only an accelerated age-related loss of filtration function, and development of symptomatic kidney failure is rare [19, 21]. However, a small minority (5–10%) of individuals with NPS develop nephrotic-range proteinuria as early as childhood or young adulthood and progress to end-stage kidney failure over variable periods of time [19, 21]. The severity of the nephropathy is extremely variable both within and between families, suggesting that genetic, epigenetic or environmental factors might modulate the severity of the nephropathy [19]. The prevalence of nephropathy has been reported to vary widely between 10–40% of individuals with nail-patella syndrome [10, 11, 19], depending maybe on the phenotypic selection of the cohort.

In nail-patella syndrome, the prognosis is determined by the nephropathy. Comparable with the human syndrome, homozygous *LMX1B* knock-out mice lack patellae and suffer from severe podocyte damage. In contrast, however, podocin and the *α*3 and *α*4 chains of collagen IV are absent in the glomeruli of *LMX1B* knock-out mice. Further studies with podocyte-specific *LMX1B* knockout mice have confirmed the importance of *LMX1B* in podocytes, as these mice apparently develop foot processes initially but lose them later on [6]. Thus, *LMX1B* is essential for the development of metanephric precursor cells into podocytes and possibly also for maintaining the differentiation of podocytes [6].

Nail-patella nephropathy includes renin-angiotensin system blockade to manage proteinuria, based on data that angiotensin antagonism reduces protein excretion through hemodynamic mechanisms and podocyte protection, slowing renal disease progression [20]. However, this therapy is palliative and is not always successful, as different mutations in LMB1X may determine the response to renin-angiotensin system blockade [21]. For patients with end-stage renal disease, as our patient, renal transplantation is the preferred renal replacement therapy, as nail-patella nephropathy does not relapse after transplantation.

In summary, the pursue of genetic causes in patients with proteinuria and a histological diagnosis of FSGS, particularly the young, are mandatory in many aspects. Not only it offers a precise cause of chronic kidney disease and potential genetic counseling but also avoids the prescription of unnecessary immunosuppression, often employed as an attempt to reduce proteinuria. In cases of proteinuria due to podocyte genetic mutations, the use of immunosuppressants has no place. Since early childhood, our patient received an unnecessary burden of toxic immunosuppression, with potential consequences for her future, added to the recently prescribed ones to manage the transplanted allograft.
